# Reports of the Sanitary Committee to the Cook Co. Medical Society

**Published:** 1858-08

**Authors:** 


### EDITORIAL.

### REPORTS OF THE SANITARY COMMITTEE TO THE COOK CO. MEDICAL SOCIETY, AUGUST 3, 1858.

### REPORT OF DR. E. L. HOLMES, FOR THE NORTH DIVISION OF THE CITY.

CHICAGO, August 3, 1858.

Your committee appointed to report upon the health of the North Division of our city, has comparatively little to offer. The season thus far has been one of remarkable health, and yet there is good reason to believe that the poor in many instances, suffering from poverty and want, have neglected to call medical aid even in cases of necessity. Undoubtedly, numerous deaths have occurred among children of the abject poor, without the knowledge of physicians. I infer this from the fact, that I have several times heard reports of such cases from people who knew the circumstances. I have also in my visits, in different sections of the North Division, seen children in a dying condition, to whom no medical assistance, as I was informed, had been called. The parents of these children, from the difficulty of gaining a support, seemed indifferent to their recovery, and even wished them to die. How far this degree of inhumanity is prevalent among the poor of our city, I am unable to judge.

During the past month, although the weather upon the whole has been delightful, abdominal diseases, especially among children, have been more prevalent and deaths more numerous. Atmospheric influences undoubtedly have much influence in producing these results, but I apprehend that neglect and inefficiency on the part of parents are the great causes of mortality among the poor children of our city. And yet if we examine into the condition of the dwellings of the poor wretches, for instance, who live near the river on the North Division, between Kinzie and Superior streets, I think we shall find other causes of disease and death. The ground is lower than in some other portions of the city, the drainage is incomplete, the contents of privies and every kind of offal remain for a long time upon the surface of the earth. It seems almost incredible that human beings can live under such circumstances. Fortunately this portion of the city will soon be improved.

I have seen and heard of numerous cases of measles and scarlatina.

My attention has been called to several very severe cases of cholera morbus. Two of these cases, I must confess, I am not disposed to regard as cholera morbus, but true cholera, perhaps of a mild form, and I think no one of experience, who had seen them in a season of epidemic, would hesitate to call them so. The first case was that of a German laborer, 45 years of age, very tall and of great apparent physical strength, who, without any known cause, was attacked in the afternoon of July 22 with severe vomiting and purging without the slightest pain. From the fact that there was no pain, patient thought he would soon be better and consequently sent for no physician. The symptoms in a couple of hours became less violent, but at four o'clock the next morning returned, with exception of the vomiting, with renewed energy. As there was still no pain, patient flattered himself that the attack would pass off without medical aid; but at four o'clock in the afternoon, after having passed more than thirty copious watery evacuations, he sent for me. I found him upon his back, nervous force almost wholly prostrate, extremities cold, lips livid, pulse feeble, cold perspiration upon the face and hands. The discharges were still very frequent, and resembled very diluted arrow-root as prepared for the sick, with something like Indian meal at the bottom of the vessel. The patient complained of nothing but thirst and weakness.

The second case was that of an Irish woman, aged 25, married, and of remarkably good constitution. Without known cause, at three o'clock, July 31, she was so violently attacked with vomiting and purging, that after throwing herself upon the bed, she had not strength enough to call the assistance of her neighbors. In a couple of hours a friend happened to call and found her in her prostrate condition. A few minutes after this on reaching the house, I found her still somewhat inclined to vomit; so weak that she could scarcely rise upon her elbow; the discharges from her bowels copious, watery and passing away involuntarily. The feather bed upon which she lay was wet through. Upon examining the sheets, I found there was no discoloration from the discharges and hardly any smell. A small quantity of the discharges collected in a vessel resembled a mixture of white Indian meal with a large proportion of water. The patient had no pain, lips blanched, extremities, however, not cold, pulse feeble, constant sighing with difficulty of breathing, complaining of intense burning thirst. Patient was evidently much frightened. This, however, could not account for the great prostration of strength, since the number and quantity of the discharges during the time I was with her, were sufficient to account for her loss of strength.

Both of these patients recovered under treatment which consisted of small doses of calomel and opium often repeated (every fifteen minutes for a couple of hours, and then every half hour for the same time), with small doses of brandy and capsicum, and injections of laudanum and starch into the rectum after every discharge; I also applied cloths wet with hot mustard water to the external surface.

In the course of a couple of hours, marked relief of symptoms appeared. In a couple of days, patients were able to leave the bed. In the last case I must admit, that the calomel less than fifteen grains produced a very slight soreness of gums.

E. L. HOLMES.

---

REPORT OF DR. D. D. WAITE, FOR THE WEST DIVISION OF THE CITY.

CHICAGO, August 3, 1858.

The undersigned Sanitary Committee for West Chicago respectfully report:

That the health of the West Division of the city, for the past month, has been unusually good for the season.

The very cool turn of weather which set in the second week of the month, succeeding the three weeks of weather so warm that the mercury stood at an average of 90 degrees, was, according to reasonable expectation, immediately followed by a general prevalence of diarrhea, cholera morbus and some dysentery. These have constituted the burthen of disease for the last three weeks. Yet in a very few cases have they proved dangerous or extremely troublesome. In my own cases, with the aid of a little calomel, I have successfully combated the endemic of the season, with a combination of morphine, tannin and quinine.

I have seen but one death from cholera infantum. This was a child of one year, who had endured the wasting disease for four or five days without intermission. The flesh and natural visage nearly gone, and in deadly collapse, at the time I was called.

Here I will state, to illustrate a fact to which I wish to call attention, that this child, in this extreme condition, was brought more than a mile in the arms of its mother, uttering an incessant moan, which plainly enough told its fate as soon as my door was opened.

Last year, I saw a number of cases of this kind among the destitute, who either died without medical aid at all, or only received it when too late to be of service. From what observations I have made, and from what I have learned last season and this, I am now satisfied that nearly one-half of all the deaths of children in the city occur from the want of timely medical aid, and the suitable nursing and surroundings of a sick person. Indeed, I know that many children of the poorest class, who live entirely outside of society and outside of common care, die without any medical aid, and sometimes, as I have also had occasion to know, while the parents were too drunk to realize their danger. These sad facts may account for the circumstance that physicians are often surprised at hearing the report of city deaths, especially of children, having seen nothing in their practice to apprise them of so large a number.

This will also in part account for the greater mortality of city than of country.

Here and there, a solitary case of intermittent or remittent fever, or, in common parlance, of ague and fever, with their tawny, muddy, or dusky-yellow visage, are occurring, like the last of the Mohicans, to remind us of a race of diseases now nearly extinct, which formerly created great havoc and terror in the early settlements of Northern Illinois and vicinity. For the management of these, little else than blue pill and quinine is needed. I will remark, that in my own practice, several severe cases of cholera morbus in adults have occurred, which, in cholera times, would certainly have been set down as such. In the case of one lady of fifty, most intolerable crampings continued, in spite of all my efforts, for some ten or twelve hours after the discharges had ceased and the circulation was in a good measure restored.

I think that ether did more than any other remedy in quieting them.

In this case, the use of opium in any form being wholly interdicted by a constitutional idiosyncrasy, I think I checked the alvine fluid (it was but water with a slight greyish tinge) with injections of sulphate of zinc. I have heard of a few fatal cases of the kind.

DANIEL D. WAITE.

---

In addition to the foregoing reports, we copy the following, which shows the entire mortality of the city for the month of July:

| | |
|---|---|
| South Division | 117 |
| West " | 81 |
| North " | 70 |
| Total | 268 |

The mortality for the same month for a series of years past is as follows:

| '48. | '49. | '50. | '51. | '52. | '53. | '54. | '55. | '56. | '57. | '58. |
|---|---|---|---|---|---|---|---|---|---|---|
| 41 | 411 | 240 | 67 | 179 | 111 | 934 | 236 | 266 | 254 | 268 |

The diseases of the month were as follows:

| | | | |
|---|---|---|---|
| Inflammation of Lungs | 9 | Convulsions | 4 |
| Inflammation of Brain | 11 | Still-born | 7 |
| Inflammation of Stomach | 2 | Erysipelas | 1 |
| Bilious Fever | 1 | Canker-Sore Mouth | 2 |
| Typhoid Fever | 1 | Consumption | 37 |
| Scarlet Fever | 23 | Debility | 3 |
| Delirium Tremens | 1 | Peritonitis | 1 |
| Ulcer of Stomach | 1 | Dropsy | 3 |
| Gastro-Enteritis | 1 | Rheumatism | 1 |
| Dysentery | 103 | Apoplexy | 1 |
| Teething | 18 | Old Age | 1 |
| Measles | 5 | Sun-stroke | 1 |
| Croup | 1 | Violent Death | 1 |
| Whooping Cough | 1 | Accident | 4 |
| Cholera Morbus | 2 | Drowned | 6 |
| Cholera Infantum | 12 | Unknown | 2 |

It will be seen that dysentery has been the chief prevailing disease for the month; furnishing almost half of the whole number of deaths. It has prevailed chiefly among children under three years of age. In most instances it has been mild and easily controled by proper treatment.

From personal observation, we think that three-fourths of the deaths from this disease have occurred among the children of the poorer classes, who are prone to attribute all affections of the bowels during the first two years of life to "teething;" and therefore neglect all medical aid until the children are irrecoverably exhausted. From various sources we have learned, that dysentery and fevers are quite prevalent in many sections of the State. But we have had no detailed account of the special characters they assume.

In this city, attacks of fever, either continued or periodical, were of rare occurrence until the third week in August. From that time to the present (Sept. 5th), we have met with febrile attacks quite frequently, and presenting some features worthy of notice. In the typhoid cases there was, during all the first week, decidedly more gastric irritation, manifested by vomiting, especially after taking either food, drink or medicine; and less intestinal disturbance than in ordinary cases. The bowels were moderately costive, and in very few instances have they become tympanitic, even in the advanced stages of the disease. Many of the patients have complained of an unusual degree of distress or heaviness through the chest and epigastrium, with constant sweating and great sense of weakness. In a few instances the sweating has commenced with the forming stage, and has continued profuse throughout the whole course of the disease, causing the skin to feel cold and clammy to the touch, and corrugated as if long macerated in water. In these cases, the pulse becomes soft and weak, the tongue moist, thirst urgent, urine scanty, and the sense of weakness extreme.

Unless a favorable change takes place during the latter part of the second week, in addition to the symptoms enumerated, the mind becomes dull and indifferent, the eyes sunken, the pulse small and quick, the lips purple or leaden color, and the breathing either very feeble or slow and sighing. In the Mercy

Hospital and among the poor in the city, I have seen three fatal cases. In one of these, hemorrhage from the bowels occurred twelve or fourteen hours before death; but in the other two, death seemed to occur from simple exhaustion of the vital properties of the tissues.

In some of the cases but little pain was felt during the whole course of the disease. But in others, the pain in the head was persistent and severe for four or five days, and followed by an unusual degree of somnolence.

In some of the cases, a copious miliary eruption appeared on the surface between the fifth and tenth day of the disease, and continued until convalescence was established. From these observations it will be seen, that the frequent periods of profuse sweating, the soft and compressible pulse, the absence of abdominal tympanitis, together with the early feelings of exhaustion, constitute the prominent or peculiar features of the present prevailing form of continued fever. And they are sufficient to indicate great organic debility, or, in other words, decided diminution of both susceptibility aud vital affinity throughout the organized structures of the body. This direct depression of the vital properties or forces causes all the primary functions, such as secretion, cell growth, muscular contraction, nerve sensibility, etc., to be feebly executed. Hence, both respiration and circulation become feeble, the skin becomes relaxed as in the advanced stage of cholera, the saline matter of the blood rapidly passes off in the copious perspiration, and the patient is in imminent danger of death, not from local congestions, inflammations, or disorganization of structures, but from simple exhaustion of vital force and consequent cessation of organic action. We might cite several cases illustrating the prevailing type of fever. But we have space for only a brief notice of three; two of which were treated in the Mercy Hospital, and the other in private practice.

Case 1. Mr. ———, a German laborer, aged about thirty-eight years, was admitted into the Mercy Hospital on the 25th of August. He had been sick about ten days. His skin was cool and wet with perspiration; his eyes dull and upper lids drooping; lips pale and tongue moist; mind incapable of being fixed long enough to answer questions correctly; pulse soft, small and 120 per minute; abdomen soft and not tympanitic, and intestinal and urinary evacuations involuntary. Although these symptoms plainly indicated a *moribund* condition of the patient, yet neither they, nor anything we could learn concerning the history of the case, indicated the existence of any special local lesions sufficient to produce a fatal result. He died on the evening of the 26th, about thirty hours after admission, apparently from entire failure of the elementary properties and functions of the system.

Case 2. Mr. B., a German, aged about forty years, was admitted into the Hospital on the 25th of August, 1858. He had been sick about ten days with continued fever, apparently mild in its character. At the time of admission, his face was moderately flushed; eyes dull; lips dry; tongue covered with a brownish coat, but not entirely dry; skin generally dry and above the natural temperature; respiration natural; pulse 100 per minute, and moderately firm; abdomen entirely free from tympanitic distension, but the bowels quite inactive, there having been no alvine evacuation during the preceding three days. He complained of much headache, sleeplessness, and occasionally a disposition to vomit. He was directed to take a powder containing calomel two grs. and bi-carb. soda five grs. every four hours until it should move the bowels.

*Aug. 26th.* Symptoms unchanged, and no alvine discharges. Ordered castor oil ℥ss., and should it operate freely, have it followed by an emulsion of oil of turpentine and tincture of opium, with two grains of quinine in each dose.

*Aug. 27th.* The castor oil operated freely yesterday, and was followed by the emulsion and quinine. The pain in the head was much relieved, otherwise the condition of the patient did not seem materially altered. Continued the same treatment with beef-tea for nourishment.

*Aug. 30th.* Found the patient this morning much more feeble. His skin was cool and very moist with perspiration, presenting on the fingers and hands a corrugated or shriveled appearance; the eyes more dull and sunken; the lips of a purplish color; tongue moist; abdomen flaccid and bowels inactive; pulse 120

per minute, small and soft; respiration unobstructed and quiet, but feeble; and mind dull or unconcerned. It was evident from the state of the skin, together with the feebleness of both respiration and circulation, that the vital forces of the patient were rapidly undergoing exhaustion. And yet, there had been no excessive evacuations or other apparent cause for such depression. To increase innervation and maintain a better degree of organic affinity, the patient was directed to take the sixteenth of a grain of strychnine in solution with four drops of nitric acid and water, every four hours, and two grains of sulphate of quinine between.

*Aug.* 31*st.* All the symptoms remain the same as yesterday, except that the circulation and respiration are both still more feeble, and the expression of the patient more sunken. Continued the strychnine and nitric acid every three hours, but omitted the quinine and gave in its place ten gr. doses of chlorate of potassa in solution; also, beef-tea, well salted, for nourishment. The object of making this change, as stated to the students visiting the Hospital at the time, was to increase the saline matter in the blood, and thereby increase its capacity for absorbing oxygen from the pulmonary organs. The purple or leaden hue of the skin, etc., plainly indicated a deficient decarbonization and oxygenation of the blood. It was evident that neither quinine nor ordinary diffusable stimulants, were calculated to remedy this elementary pathological condition. But if a greater quantity of oxygen could be introduced into the blood, it would do more to re-excite and sustain the failing organic sensibility and capillary action, than all other agencies with which we are acquainted. Hence, we ordered the chlorate of potassa as medicine and the chloride of sodium as condiment for the animal broth. The result in this case fully justified our theory; for on the following day we found every symptom of the patient improved. And by continuing the treatment four days, convalescence was fully established. During the last few weeks we have had occasion to prescribe the chlorate of potassa in several cases of continued fever presenting similar symptoms with the patient just alluded to and with uniform advantage. The following affords a striking example:

CASE 3. Mr. M., a young man, native of Ireland, had been sick with continued fever about seven days, and under the care of an intelligent physician. On Tuesday, a messenger called on us saying, that the young man had been given up by his physician, and he was very anxious that we should see him. Knowing that there was very little reliance to be placed on the representations of that class of people, we did not answer the call that day. The next it was repeated with still more urgency, and we visited the patient. Found him occupying a dorsal position in bed; the head thrown back; the eyes turned upward, with drooping of the upper eyelids; a leaden hue of the lips; skin generally cool, covered with perspiration, and of a color indicating deficiency of capillary circulation; bowels inactive and abdomen flaccid; tongue and mouth moist; pulse small, soft and 115 per minute; respiration easy but feeble, and mind very dull. The attendants had not been able to make him "know anything" during the last twenty-four hours, and it was with difficulty that we induced him to partially protrude his tongue. The urine passed involuntarily, and the last fæcal evacuation was not controled by the patient. Finding no evidence of local structural lesions, and believing the condition of this patient essentially dependent on the same loss of organic sensibility or susceptibility, with consequent diminution of capillary action and æration of the blood, as described in reference to Case 2, we ordered for him the following:

℞ Chlorate of potassa, ʒij.

Mucilage of gum Arabic, ℥iv.

Mix, and give a table-spoonful every two hours; also, ten drops of chloroform, in sweetened water, between each of these doses. For nourishment, we directed beef-tea, well salted, or milk. The treatment was faithfully carried out, and in less than twenty-four hours he became sufficiently conscious to answer questions correctly, the clammy sweat disappeared, and his pulse became slower and more full. The same treatment was continued, only the medicine given at longer intervals. In two days more he had recovered full possession of his mental faculties, and control of his evacuations, with a pulse 90 per minute and soft, and some relish for nourishment. The chloroform was now omitted, and the chlorate of potassa continued every three hours. No other medicines have been administered, and the patient is now, fifteen days from the commencement of the attack, convalescent.

In the commencement of this article we used the word typhoid; but it is evident that the continued fevers we have met with during the last three weeks have partaken much more of the nature of *typhus* than of typhoid fever, as technically defined by medical writers. Since the middle of August, attacks of periodical fever have been more frequent than usual. Most of the cases that have come under my observation have been simple, and easily interrupted in their course by ordinary anti-periodic doses of quinine. But a few cases have exhibited a decidedly pernicious and dangerous character. The following case will illustrate these attacks better than any general description:

CASE 4. Mr. R., a native of Ireland, aged twenty years, was admitted into the Mercy Hospital, Sept. 1st, 1858. He had been sick five days. Said he had had chills and fever every day, the cold stage occurring in the evening. At present, his countenance is dull, lips slightly bluish or leaden color, tongue moist but coated, bowels inactive, stomach irritable, with disposition to reject drinks, and complains of a sense of oppression or distress in the epigastrium. His skin is nearly natural in temperature and moisture. Fearing that quinine, if given immediately, would be rejected on account of the irritability of the stomach, we directed for him three or four small doses of calomel and morphine, to be followed by castor oil sufficient to open the bowels. Before the time came, however, to take the oil, his next paroxysm came on. The cold stage was characterized by great depression; a small, frequent and feeble pulse; a purplish or leaden hue of the skin; great restlessness and anxiety, but with very little muscular agitation or trembling. The febrile reaction was slow and imperfect, and the sweating stage slight.

As soon as the character of this paroxysm had been observed, the patient commenced taking sulph. of quinine four grs., with pulv. opii one and a half grs., every three hours. The medicine was taken faithfully throughout the day and was not rejected by vomiting. Still, at the usual hour in the evening, another paroxysm came on, with symptoms of the most dangerous character. We made an extra visit to the Hospital near nine o'clock in the evening, and found him with lips and face of a dingy hue; the skin everywhere cold and covered with a clammy moisture; the pulse 142 per minute, small and feeble; the respiration irregular, intermittent and sighing; great restlessness and sense of oppression in the chest and epigastric region, with occasional efforts to vomit. He had been in this state nearly three hours. We immediately discontinued the quinine and opium, ordered a strong sinapism of mustard to the dorsal spine, and internally ten grs. of chlorate of potassa every hour, and ten gtts. of chloroform between, until indications of reaction were seen, when the intervals between the doses should be lengthened. The next morning found the patient quiet, skin warm and dry, pulse 95 per minute, general feeling comfortable though weak. Directed the chlorate of potassa and chloroform to be continued alternately two hours apart; and as the bowels had not been moved during the last three or four days, three powders, each containing quinine three grs. and calomel five grs., were given at intervals of four hours, to be followed by castor oil if they did not operate. The bowels were freely moved after the oil on the morning of the 4th, and the patient continued much improved. The chloroform was omitted, and the chlorate of potassa alone continued, with animal broth for nourishment. The patient had no further paroxysms, and is now quite well.

In three other strongly-marked cases of pernicious intermittents, we have prescribed the same remedies with the same happy results. Sometimes, we combine the chloride of sodium with the chlorate of potassa. It has seemed to me that the fevers of the present season, whether periodical or continued, present a strong typhus tendency. And the principal object of the foregoing observations is to call the attention of our readers to this feature, and awaken thought as to the best mode of counteracting it. Will some of our readers in those country districts where fevers are prevalent, favor us with an account of their special characteristics?

